# Rounding of low serum creatinine levels and consequent impact on accuracy of bedside estimates of renal function in cancer patients

**DOI:** 10.1038/sj.bjc.6601641

**Published:** 2004-03-02

**Authors:** M J Dooley, S Singh, D Rischin

**Affiliations:** 1 Pharmacy Department, Peter MacCallum Cancer Centre, St Andrew's Place, East Melbourne 3002, Australia; 2 Department of Pharmacy Practice, Victorian College of Pharmacy, Monash University, 381 Royal Parade, Parkville 3052, Australia; 3 Department of Haematology and Medical Oncology, Peter MacCallum Cancer Centre, St Andrew's Place, East Melbourne 3002, Australia

**Keywords:** glomerular filtration rate, Cockcroft and Gault, renal function, creatinine clearance, serum creatinine

## Abstract

To compare glomerular filtration rate measured by technetium-99m ([Tc^99m^]) DTPA clearance with estimated creatinine clearance (CrCl) (Cockcroft and Gault (C&G) method) in patients with serum creatinine (Scr) levels <0.06 mmol l^−1^, and determine the effect of rounding serum creatinine to 0.06 mmol l^−1^. Patients with serum creatinine values <0.06 mmol l^−1^ at the time of [Tc^99m^] clearance determination were identified. Creatinine clearance was calculated by the C&G method using both actual and rounded Scr values. A total of 419 adults had GFR measured by technetium-99m diethyl triamine penta-acetic acid ([Tc^99m^] DTPA) clearance. Out of this group, 26 patients had a serum creatinine value <0.06 mmol l^−1^. The C&G estimates of renal function using actual serum creatinine resulted in an overall overestimation of 12.9% when compared to [Tc^99m^] DTPA clearance. When the value of serum creatinine was rounded to 0.06 mmol l^−1^, the formula underestimated renal function by −7.0%. Analysis of estimated creatinine clearance for different levels of renal function showed significant differences to [Tc^99m^] DTPA clearance. Rounding up of serum creatinine to 0.06 mmol l^−1^ improved the predictive ability of the C&G method for the patients with [Tc^99m^] DTPA clearance ⩽100 ml min^−1^, but worsened the effect in those >100 ml min^−1^. This work indicates that when bedside estimates of renal function are calculated using the C&G formula actual Scr should be used first to estimate CrCl. If the resultant CrCl is ⩽100 ml min^−1^, then the Scr should be rounded up to 0.06 mmol l^−1^ and CrCl recalculated. Further assessment of this approach is warranted in a larger cohort of patients.

An assessment of renal function is required when determining the dosage of cytotoxic drugs that are renally excreted. Ideally, a method for estimating glomerular filtration rate (GFR) is required that may be performed at the bedside. Any such estimate needs to be accurate, convenient and inexpensive, and consequently should be a noninvasive formula-based method, which does not require multiple blood samples or tedious urine collection. Creatinine clearance (CrCl) measurement, through 24-h urine collection, has been used to estimate renal function; however, the reliability of this method is very much dependent on an accurate and complete urine collection, and is therefore frequently unsuitable (Davila and Gardner 1987; McDermott *et al*, 1987; Chambers *et al*, 1990; Luke *et al*, 1990; Robinson *et al*, 1990; Tsubaki *et al*, 1993; Millward *et al*, 1996). Various equations and nomograms have been developed to estimate CrCl from serum creatinine (Scr) concentration (Jelliffe, 1973; Cockcroft and Gault 1976; Martin *et al*, 1998; Wright *et al*, 2001).

In practice, some patients are encountered who have very low Scr. When such low results are incorporated into various formulae to estimate CrCl, there is concern that this may result in an inaccurate prediction. It has been a common clinical practice to round up the Scr level of these patients when using bedside estimates of renal function (Bertino, 1993; Smythe *et al*, 1994). In addition, various clinical trials have stipulated this requirement, for example, the current Phase III GOG clinical trial in first-line treatment of ovarian cancer (GOG182). The most common scenario is to arbitrarily round Scr levels <0.06 to 0.06 mmol l^−1^ and incorporate this into the Cockcroft and Gault (C&G) formula to estimated renal function. It is recognised by clinicians that this is a subjective decision. One of the reasons for the practice of ‘rounding up’ is concern with respect to the potential overestimation of renal function if very low Scr is used. Using an overestimate of renal function could result in the potential of overdosing of patients with cytotoxic chemotherapy agents, such as carboplatin, especially in patients with low Scr postoperatively who may be malnourished.

The validity of this ‘rounding up’ approach has not been determined. An accurate measurement of GFR is possible by measuring the clearance of the radiolabelled isotopes such as technetium-99m diethyl triamine penta-acetic acid ([Tc^99m^] DTPA) and chromium 51-ethylene diamine tetra-acetic acid (Cr^51^ EDTA) (Rehling *et al*, 1984; Fawdry *et al*, 1985; Peters, 1991; Millward *et al*, 1996). The aim of this study was to compare measured GFR [Tc^99m^] DTPA with estimated CrCl –calculated by the C&G method in patients with Scr levels <0.06 mmol l^−1^, and determine implication of rounding Scr to 0.06 mmol l^−1^.

## MATERIALS AND METHODS

This was a retrospective study of adult patients with cancer who had GFR measured by [Tc^99m^] DTPA clearance at the Peter MacCallum Cancer Centre between March 2000 and June 2003. Patients with Scr values <0.06 mmol l^−1^ at the time of [Tc^99m^] DTPA clearance determination were identified. Height and actual body weight were measured. Age and gender were recorded.

GFR was determined by [Tc^99m^] DTPA clearance (Dooley *et al*, 2002). [Tc^99m^] DTPA was prepared 30–60 min prior to injection using fresh elute and a current DTPA kit (Amersham International formulation). Instant thin layer chromatography was performed on all DTPA preparations approximately 30 min after reconstitution of the kit and at the time of dose administration. Radioactivity was sampled in a Well scintillation counter to confirm labelling efficiency of greater than 98%. [Tc^99m^] DTPA (400 MBq) was administered via a three-way tap and cannula to enable correlation with renal imaging. A 10 ml sodium chloride 0.9% flush per dose ensured no dose residue in any of the apparatus. Dose apparatus and injection site were checked for dose residue using a scintillation probe. Blood samples (10 ml) were taken at baseline and at 2, 3 and 4 h postinjection. Plasma was separated and counts obtained. The clearance of [Tc^99m^] DTPA was calculated from a single exponential derived from the blood samples between 2 and 4 h after injection. The GFR was calculated without correction for body surface area (BSA).

Scr was measured using an alkaline picrate kinetic method, with Roche Diagnostic Hitachi 912 reagent. Creatinine clearance was then calculated by the C&G method using Eq. (1) from both actual Scr value and then rounded to 0.06 mmol l^−1^. Body surface area (Mosteller, 1987) and body mass index (BMI) (World Health Organization, 2000) were calculated using Eqs. (2) and (3).


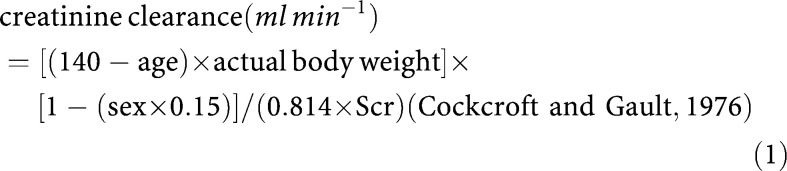


where the actual body weight is measured in kilograms, Scr is measured in micromoles per litre, age in years and sex=0 (males) or 1 (females).


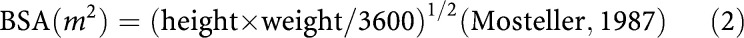


where BSA is measured in square metres, height in centimeters and body weight in kilograms


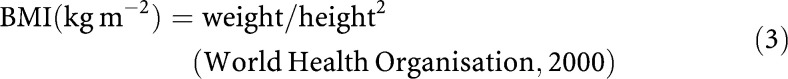


where weight is measured in kilograms and height in metres.

The difference between the measured GFR and estimated CrCl were examined to determine whether prediction error was independent of measurement magnitude. Analyses of differences were used to determine bias and precision. Bias was assessed by mean percentage error (MPE), calculated as the percentage difference between the estimated CrCl and measured GFR. A positive bias indicates overestimation of GFR and a negative bias indicates underestimation. Paired *t*-test was used to compare the actual and corrected CrCl at *α*=0.05 with [Tc^99m^] DTPA measured GFR.

## RESULTS

A total of 419 adults had GFR measured by [Tc^99m^] DTPA clearance during the period under study (March 2000 and June 2003). In all, 26 adult patients in this group were found to have Scr value of <0.06 mmol l^−1^. There were 11 males and 15 females. Patient demographics are detailed in Table 1

**Table 1:** Demographic characteristics of patients (*n*=26) with Scr values <0.06 mmol l^−1^

	**All (*n*=26) mean (range)**	**Females (*n*=15) mean (range)**	**Males (*n*=11) mean (range)**
Height (cm)	166 (152–187)	161 (152–170.5)	173 (153–187)
Weight (kg)	62 (32–100)	64.0 (44–100)	60 (32–81)
Age (years)	57 (21–84)	58 (40–76)	57 (21–84)
BSA (m^2^)	1.69 (1.17–2.14)	1.68 (1.36–2.14)	1.70 (1.17–1.97)
BMI (kg m^−2^)	22.5 (13.7–36.8)	24.6 (17.3–36.8)	20.0 (13.7–27.1)

Scr=serum creatinine; BSA=body surface area; BMI=body mass index.

.

Results of measured GFR and estimated CrCl are detailed in Table 2

**Table 2:** Calculated CrCl values using actual and rounded Scr values compared to [Tc^99m^] DTPA clearance in patients with Scr <0.06 mmol l^−1^

		**Mean±s.d. (ml min^−1^)**	**Range (ml min^−1^)**	**MPE (%)**	***P*-value**
[Tc^99m^] DTPA	All	111±46	45–256		
	⩽100 ml min^−1^	77±14	45–96		
	>100 ml min^−1^	140±45	103–256		

C&G	All	117±38	55–207	12.9	0.352
	⩽100 ml min^−1^	98±28	55–152	29.2	0.024
	>100 ml min^−1^	135±38	86–207	−0.1	0.631

C&G (with rounded Scr)	All	97±30	46–172	−7.0	0.029
	⩽100 ml min^−1^	82±23	46–127	7.9	0.543
	>100 ml min^−1^	110±29	72–172	−18.9	0.003

**Figure 1:**
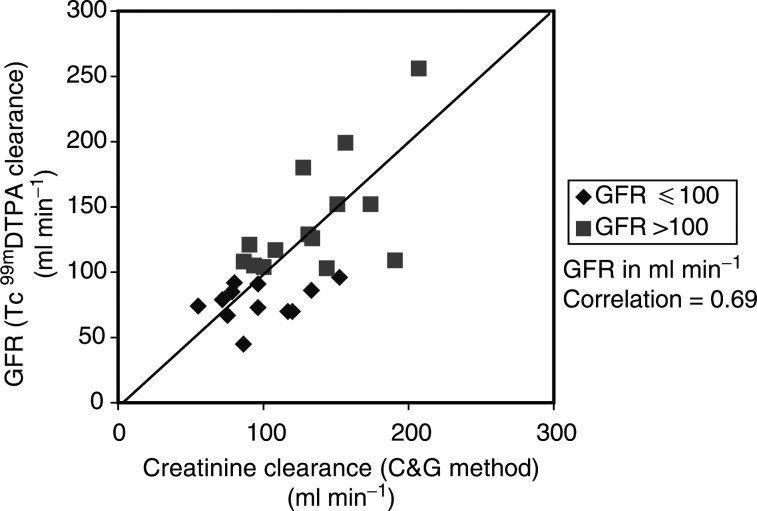
[Tc^99m^] DTPA clearance and CrCl estimated by the C&G method using actual Scr values.

CrCl=creatinine clearance; Scr=serum creatinine; [Tc^99m^] DTPA=technetium-99m diethyl triamine penta-acetic acid; C&G=Cockcroft and Gault; MPE=mean percentage error.

and shown graphically in Figures 1

, 2

**Figure 2:**
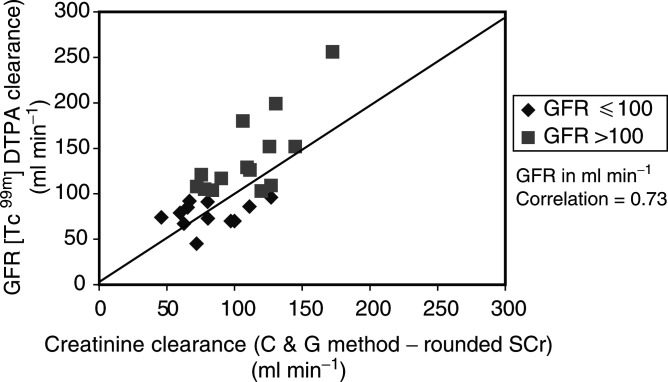
[Tc^99m^] DTPA clearance and CrCI estimated by the C&G method using rounded Scr values.

, 3

**Figure 3:**
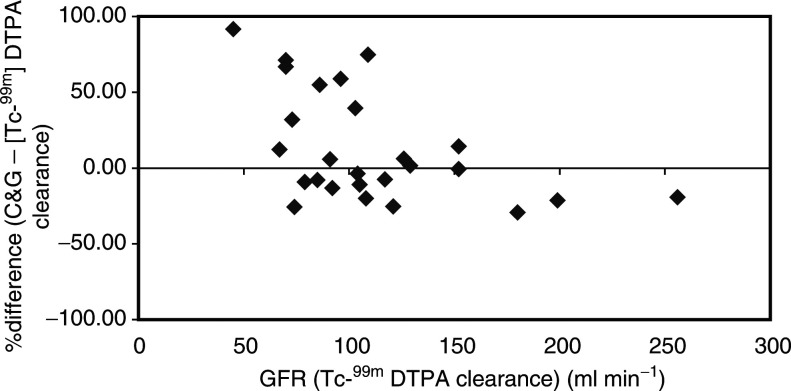
Percentage difference between [Tc^99m^] DTPA clearance and CrCI estimated by the C&G method using actual Scr values.

and 4

**Figure 4:**
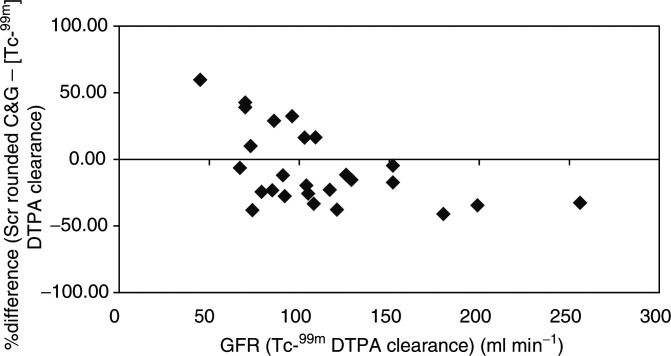
Percentage difference between [Tc^99m^] DTPA clearance and CrCI estimate by the C&G method using Scr rounded to 0.06 mmol l^−1^.

. There were 25 patients with Scr of 0.05 mmol l^−1^ and one patient with Scr of 0.04 mmol l^−1^.

The C&G estimates of renal function using actual Scr resulted in an overall overestimation of 12.9% when compared to [Tc^99m^] DTPA clearance. When the value of Scr was rounded to 0.06 mmol l^−1^, the formula underestimated renal function by −7.0%.

The analysis of estimated CrCl for different levels of renal function showed significant differences to [Tc^99m^] DTPA clearance. Patients with [Tc^99m^] DTPA clearance ⩽100 ml min^−1^ had CrCl estimated that were less predictive than those >100ml min^−1^. Rounding up of Scr to 0.06 mmol l^−1^ improved the predictive ability of the C&G method for the patients with [Tc^99m^] DTPA clearance ⩽100 ml min^−1^, but worsened the effect in those with >100 ml min^−1^.

Four patients (15.4%) were underweight (BMI <18.5 kg m^−2^), 15 (57.7%) patients were normal weight (BMI 18.5–25 kg m^−2^) and seven (26.9%) patients were overweight (BMI <25 kg m^−2^). The mean C&G and [Tc^99m^] DTPA clearance in BMI categories are detailed in Table 3

**Table 3:** Calculated CrCl estimates using actual and rounded Scr values in BMI categories in patients with Scr <0.06 mmol l^−1^

	**BMI (kg m^−2^)**	**Mean±s.d. (ml min^−1^)**	**Range (ml min^−1^)**	**MPE (%)**	***P*-value**
[Tc^99m^] DTPA	<18.5	102±19.9	74–121		
	18.5–25	103±41.2	45–199		
	>25	133±63.0	70–256		

C&G	<18.5	81±22.9	48–100	−21.0	0.04
	18.5–25	115±34.7	75–191	20.0	0.25
	>25	146±36.2	96–207	20.0	0.34

C&G (with rounded Scr)	<18.5	72±10.2	59–84	−27.7	0.03
	18.5–25	93±24.8	63–130	−2.3	0.26
	>25	122±30.1	80–172	0.1	0.46

CrCl=creatinine clearance; Scr=serum creatinine; BMI=body mass index; [Tc^99m^] DTPA=technetium-99m diethyl triamine penta-acetic acid; C&G=Cockcroft and Gault; MPE=mean percentage error.

.

The *r*^2^ (least-squares line) for the [Tc^99m^] DTPA clearance measurement ranged from 0.9791 –to 0.9999 (mean: 0.9956, s.d.: 0.0061).

## DISCUSSION

A small number of patients present with Scr levels <0.06 mmol l^−1^, approximately one in 16 patients in this study population. This study evaluated the common practice of arbitrarily rounding Scr in these patients to 0.06 mmol l^−1^. There is no published evidence of the frequency in practice outside anecdotal reports and as specified requirements in assessment of renal function in clinical trials. The results of this study indicate that this practice does not result in a reduction in the precision of the C&G formula to estimate CrCl for patients with GFR ⩽100ml min^−1^, it actually improves the estimate nearer to the true GFR. Conversely, for those patients with a GFR >100 ml min^−1^ rounding Scr up to 0.06 mmol l^−1^ reduced the precision of the estimate. Consequently, for patients with an Scr less than 0.06 mmol l^−1^ round upwards should only be applied to those whose estimated CrCl is less than ⩽100 ml min^−1^. When this rule is applied, the difference between actual GFR and estimated GFR for GFR ⩽100 ml min^−1^ was 7.9%.

There are a number of reasons for very low levels of Scr, primarily relating to either low level of production or a high clearance. The rounding up practice is based on the assumption of the former rather than the latter. The rate of creatinine production is related to age, sex and body weight, and these are used to scale relationships between CrCl and Scr (Bjornsson *et al*, 1983). Serum creatinine increases with age, more so in women and is at all ages higher in men (Kesteloot and Joossens, 1996). In patients suffering from concurrent hepatic diseases, the estimation of CrCl tends to result in substantial overprediction of observed CrCl. A diminished rate of creatinine production in patients with hepatic disease is likely the explanation for this anomaly (Cocchetto *et al*, 1983).

There are a number of equations and nomograms to estimate CrCl from Scr concentration (Jelliffe, 1973; Cockcroft and Gault, 1976; Bjornsson *et al*, 1983; Martin *et al*, 1998; Wright *et al*, 2001). In this study, the C&G formula was applied as this is the most common formula in routine practice. A number of groups have assessed the accuracy of the C&G approximation in a variety of clinical settings. These assessments have usually been compared to CrCl, determined by the 24-h urine collection (Chow and Schweizer, 1985; Pesola *et al*, 1993; Cochran and St John, 1993). There have also been a number of comparisons of the C&G approximation with [Tc^99m^] DTPA, Cr^51^ EDTA and other direct measures of GFR (Davila and Gardner, 1987; Robinson *et al*, 1990; Kesteloot and Joossens, 1996; Millward *et al*, 1996; Poole *et al*, 2002). These assessments have almost uniformly concluded that the C&G approximation underestimates GFR for normal and moderately reduced levels of renal function.

Our results show that when low levels of Scr are rounded to 0.06 mmol l^−1^, it actually improves the predictive ability of the formula for patients with GFR ⩽100 ml min^−1^. These results are supported by a pharmacokinetic study performed in patients with varying renal function who were treated with gentamicin daily (Kirkpatrick *et al*, 1999). The clearance of gentamicin was more accurately predicted when low levels of Scr were also capped at 0.06 mmol l^−1^. In another, albeit much smaller study of critically ill patients, the incorporation of higher corrected Scr value led to a better prediction of CrCl in cachectic patients (Robert *et al*, 1993). This study differed in some ways to ours in that the criterion standard was inulin clearance. However, other authors have concluded that rounding of lower Scr concentrations to a higher value before applying in C&G equation results in poorer correlation with direct measures (Bertino, 1993; Smythe *et al*, 1994). A potential reason for the latter conclusion may be that these papers utilised 24-h urine collection measurements and rounding to 0.085 and 0.088 mmol l^−1^, respectively.

There were only four patients in this study who were classified as underweight and hence could be expected to be low producers of muscle creatinine. All these patients had a normal GFR. In these patients, rounding of Scr resulted in further reduction in the precision of their GFR estimates. It is not possible to draw a direct conclusion due to the small number; however, concerns that overdosing of these individuals could occur with this method and are not warranted. It is more likely that lower doses of renally excreted drugs, such as carboplatin, would be given in these cases as renal function was underestimated. In the overweight patients, rounding resulted in improved mean values of CrCl compared with uncorrected low Scr, suggesting that in such patients low Scr is related to its enhanced clearance as their mean [Tc^99m^] DTPA clearance is above the normal value of 120 ml min^−1^.

There are a number of points that need to be acknowledged when considering these results. The use of the Jaffe's method of Scr measurement has been shown to influence the accuracy of the estimate; however, the methodology used in the laboratory for this study has been validated against enzymatic methods and showed less than 1.2% variability, indicating minimal influence on the results (Dooley *et al*, 2002). There was a fairly small number of subjects included, however, as stated earlier, patients meeting the criteria for assessment represent a small subset of the patient population. This study applied direct measurement of GFR, namely [Tc^99m^] DTPA clearance, and the conclusions are not influenced by limitation of comparison to CrCl estimations from 24-h urine collections. The C&G formula was utilised and even though there are other bedside formulae available, all have similar significant limitations (Poole *et al*, 2002)]. It must be noted that when any of these bedside estimates are to be used for dosage adjustment of renally eliminated drugs, then the clinician must have an appreciation of the inherent limitations. When an accurate estimate of GFR is required, then clearance should be measured using a method such as [Tc^99m^] DTPA or Cr^51^ EDTA.

This study compared measured GFR ([Tc^99m^] DTPA) with estimated CrCl in patients with Scr levels <0.06 mmol l^−1^. This work indicates that when bedside estimates of renal function are calculated using the C&G formula, then actual Scr should be used first to estimate CrCl. If the resultant CrCl is ⩽100 ml min^−1^, then the Scr should be rounded up to 0.06 mmol l^−1^ and CrCl recalculated. Further assessment of this approach is warranted in a larger cohort of patients.

## References

[bib1] Bertino Jr JS (1993) Measured versus estimated creatinine clearance in patients with low serum creatinine values. Ann Pharmacother 27: 1439–1442 8305771 10.1177/106002809302701203

[bib2] Bjornsson TD, Cocchetto DM, McGowan FX, Verghese CP, Sedor F (1983) Nomogram for estimating creatinine clearance. Clin Pharmacokinet 8: 365–369 6617044 10.2165/00003088-198308040-00007

[bib3] Chambers JT, Chambers SK, Schwartz PE (1990) Correlation between measured creatinine clearance and calculated creatinine clearance in ovarian cancer patients. Gynecol Oncol 36: 66–68 2295454 10.1016/0090-8258(90)90110-7

[bib4] Chow MS, Schweizer R (1985) Estimation of renal creatinine clearance in patients with unstable serum creatinine concentrations: comparison of multiple methods. Drug Intell Clin Pharm 19: 385–390 4006730 10.1177/106002808501900513

[bib5] Cocchetto DM, Tschanz C, Bjornsson TD (1983) Decreased rate of creatinine production in patients with hepatic disease: implications for estimation of creatinine clearance. Ther Drug Monit 5: 161–168 6879639 10.1097/00007691-198306000-00002

[bib6] Cochran M, St John A (1993) A comparison between estimates of GFR using [99m]TcDTPA clearance and approximation of Cockcroft and Gault. Aust NZ J Med 23: 494–497 10.1111/j.1445-5994.1993.tb01836.x 8297280

[bib7] Cockcroft DW, Gault MH (1976) Prediction of creatinine clearance from serum creatinine. Nephron 16: 31–41 1244564 10.1159/000180580

[bib8] Davila E, Gardner LB (1987) Clinical value of the creatinine clearance before the administration of chemotherapy with cisplatin. Cancer 60: 161–164 3594352 10.1002/1097-0142(19870715)60:2<161::aid-cncr2820600206>3.0.co;2-v

[bib9] Dooley MJ, Poole SG, Rischin D, Webster LK (2002) Carboplatin dosing gender bias and inaccurate estimates of glomerular filtration rates. Eur J Cancer 38: 44–51 11750838 10.1016/s0959-8049(00)00455-x

[bib10] Fawdry RM, Gruenewald SM, Collins LT, Roberts AJ (1985) Comparative assessment of the techniques for estimation of glomerular filtration rate with 99mTc-DTPA. Eur J Nucl Med 11: 7–12 3899658 10.1007/BF00440953

[bib11] Jelliffe RW (1973) Creatinine clearance: bedside estimate. Ann Intern Med 79: 604–605 4748282 10.7326/0003-4819-79-4-604

[bib12] Kesteloot H, Joossens JV (1996) On the determinants of the creatinine clearance: a population study. J Hum Hypertension 10: 245–249 8736456

[bib13] Kirkpatrick CMJ, Duffull SB, Begg EJ (1999) Pharmacokinetics of gentamicin in 957 patients with varying renal function dosed once daily. Br J Clin Pharmacol 47: 637–643 doi: 10.1046/j.1365–2125.1999.00938 10383541 10.1046/j.1365-2125.1999.00938.x PMC2014263

[bib14] Luke DR, Halstenson CE, Opsahl JA, Matzke GR (1990) Validity of creatinine clearance estimates in the assessment of renal function. Clin Pharmacol Ther 48: 503–508 2225710 10.1038/clpt.1990.186

[bib15] Martin L, Chatelut E, Boneu A, Rostaing L, Roussilhes C, Caselles O, Canal P (1998) Improvement of the Cockcroft–Gault equation for predicting glomerular filtration in cancer patients. Bull Cancer 85: 631–636 9752271

[bib16] McDermott DF, Galindo A, Sherman RL, Jaffe EA, Coleman M, Pasmantier MW (1987) Inadequacy of predicted creatinine clearance as guide to chemotherapy. Cancer Treat Rep 71: 1067–1069 3677111

[bib17] Millward MJ, Webster LK, Toner GC, Bishop JF, Rischin D, Stokes KH, Johnston VK, Hicks R (1996) Carboplatin dosing based on measurement of renal function – experience at the Peter MacCallum Cancer Institute. Aust NZ J Med 26: 372–379 10.1111/j.1445-5994.1996.tb01925.x 8811211

[bib18] Mosteller RD (1987) Simplified calculation of body-surface area. N Eng J Med 317: 1098 10.1056/NEJM198710223171717 3657876

[bib19] Pesola GR, Akhavan I, Madhu A, Shah NK, Carlon GC (1993) Prediction equation estimates for creatinine clearance in the intensive care unit. Intern Care Med 19: 39–43 10.1007/BF01709276 8440797

[bib20] Peters AM (1991) Quantification of renal haemodynamics with radionucleotides. Eur J Nucl Med 18: 274–286 2070806 10.1007/BF00186653

[bib21] Rehling M, Moller ML, Lund JO, Trap-Jensen J (1984) Simultaneous measurement of Tc-99mDTPA, Cr-51 EDTA and inulin in man. Clin Sci 66: 613–619 10.1042/cs0660613 6423339

[bib22] Poole SG, Dooley MJ, Rischin (2002) A comparison of bedside renal function estimates and measured glomerular filtration rate (Tc^99m^DTPA clearance) in cancer patients. Ann Oncol 13: 949–955 12123341 10.1093/annonc/mdf236

[bib23] Robert S, Zarowitz BJ, Peterson EL, Dumler F (1993) Predictability of creatinine clearance estimates in critically ill patients. Crit Care Med 21: 1487–1495 8403957 10.1097/00003246-199310000-00016

[bib24] Robinson BA, Frampton CM, Colls BM, Atkinson CH, Fitzharris BM (1990) Camparison of methods of assessment of renal function in patients with cancer treated with cisplatin, carboplatin or methotrexate. Aust NZ J Med 20: 657–662 10.1111/j.1445-5994.1990.tb00395.x 2285382

[bib25] Smythe M, Hoffman J, Kizy K, Dmuchowski C (1994) Estimating creatinine clearance in elderly patients with low serum creatinine concentrations. Am J Hosp Pharm 51: 198–204 8160670

[bib26] Tsubaki T, Goodwin S, Leader WG, Chandler MHH (1993) Estimation of creatinine clearance in patients with gynecologic cancer. Clin Pharmacol 12: 685–690 8306567

[bib27] World Health Organization (2000) Obesity: Preventing and Managing the Global Epidemic Report of a WHO Consultation. Geneva: World Health Organization 11234459

[bib28] Wright JG, Boddy AV, Highley MS, Fenwick J, McGill A, Calvert AH (2001) Estimation of glomerular filtration rate in cancer patients. Br J Cancer 84: 452–459 11207037 10.1054/bjoc.2000.1643 PMC2363765

